# Details on Ethical Review and Data Collection Added to Methods Section

**DOI:** 10.1001/jamanetworkopen.2023.31967

**Published:** 2023-08-30

In the Original Investigation titled “Mental Health Morbidities and Time to Cancer Diagnosis Among Adults With Colon Cancer in England,”^1^ published October 31, 2022, details regarding the study protocol, ethics review, approval, and data collection were added to the Methods section.^1^
